# #ChoosePsychiatry #the60secondchallenge – a thematic analysis of videos published on Twitter about reasons for choosing psychiatry

**DOI:** 10.15694/mep.2018.0000261.1

**Published:** 2018-11-16

**Authors:** Jonathan Cunliffe, Helen Hargreaves, William Melton, Reem Abed

**Affiliations:** 1 The University of Sheffield; 2 Northumberland; 3 Sheffield Health and Social Care NHS Foundation Trust

**Keywords:** medical education, psychiatry, recruitment, twitter, video, social media, #choosepsychiatry, #the60secondchallenge, undergraduate, postgraduate, thematic analysis, career, specialty recuitment, specialty choice

## Abstract

This article was migrated. The article was marked as recommended.

Aims and Method

#ChoosePsychiatry is the Royal College of Psychiatrist’s social media campaign aimed at medical students and foundation doctors to encourage recruitment into psychiatry.  This study explored the reasons given for choosing psychiatry in videos uploaded by psychiatrists to Twitter alongside the campaign, through the use of thematic analysis.

Results

Each psychiatrist gave different reasons for choosing psychiatry but four main themes were identified: prior experience of psychiatry, career factors, patient factors and knowledge factors.  Both undergraduate and postgraduate experiences were influential in choosing psychiatry.  In particular, perceived job satisfaction, an opportunity to deliver holistic care and a sense of making a difference were also influential in choosing psychiatry.

Clinical Implications

Findings here support efforts to improve the undergraduate experience and to increase foundation posts, as well as maintaining some of the current key messages of the campaign.  However, whilst engagement with the videos on Twitter was observed, further consideration of the most appropriate social media platform to ensure engagement with the target audience is needed.

## Introduction

Recruitment to psychiatry remains problematic, with only 82% of core training posts filled in 2018 ( Henfrey, 2015; Health Education England North West, 2018). The College’s current response to psychiatry’s long-standing recruitment issues is a three-year recruitment strategy. The objectives of this strategy are to target stigma, raise awareness of the clinical needs of patients and opportunities within the specialty, and to increase engagement ( The Royal College of Psychiatrists, 2017c).  Medical students and foundation doctors are identified as specific target groups within this strategy given that time spent in undergraduate medical education and foundation training are nodal points in a doctor’s career - when attitudes and beliefs about psychiatry are likely to be open to change ( Mukherjee, Maier and Wessely, 2013).

#ChoosePsychiatry is a largely social media focused campaign led by the Royal College of Psychiatrists to encourage recruitment into psychiatry.  It is aimed at attracting medical students and foundation doctors through key messages, highlighting: the need for more psychiatrists in the NHS; the depth of relationships with patients; the opportunity afforded to offer holistic care; the rewarding challenges faced; job satisfaction amongst staff; and a real impact on saving lives ( The Royal College of Psychiatrists, 2017b). In October 2017, alongside this campaign, a number of psychiatrists posted videos on Twitter undertaking ‘#the60secondchallenge’. In these videos they spoke for sixty seconds about why they chose psychiatry as a specialty, with an intention to promote and enthuse others to do the same. Recruitment to psychiatry increased in 2018, compared to 2017, and it is possible that the #ChoosePsychiatry campaign may have contributed to this ( Rimmer, 2018).

This study explored the reasons given for choosing psychiatry in the videos uploaded to Twitter. The aim is to contribute to the understanding of why psychiatrists chose psychiatry and how twitter is used as a medium for encouraging recruitment to psychiatry, seeking positive reasons to help enhance and inform the #ChoosePsychiatry campaign.

## Methods

Videos were identified through searching the twitter hashtags #the60secondchallenge and #ChoosePsychiatry in April 2018. Hashtags enable content to be marked by the user as being related to a particular topic. Thirteen videos were identified: all were included for analysis and were transcribed by the research team. Replies to the videos were searched for any other videos that had been uploaded but not connected to the hashtags; none were identified. Thematic analysis was used to identify key themes and subthemes in the videos, exploring the reasons people gave for choosing a career in psychiatry ( Guest, MacQueen and Namey, 2012). Themes were identified individually and were then discussed between researchers to achieve consensus on the final themes and sub themes. Once these had been agreed all the videos were reviewed by both analysts to assess the number of times each sub theme occurred.

## Results/Analysis

Each psychiatrist gave different reasons for choosing psychiatry. Within their responses thematic analysis identified 4 major themes: prior experience of psychiatry, career factors, patient factors and knowledge factors. Within these themes were 21 subthemes, which are summarised below in Table 1.

**Table 1.** Results of thematic analysis

Theme	Sub theme	Number of examples
Prior Experience	Positive undergraduate experience	7
	Positive postgraduate experience	2
	Previous positive views	2
	Previous negative views	2
	Role models	2
Career	Job satisfaction	8
	Job variety	4
	No regrets	4
	Teamwork	4
	Teaching	3
	Supplementary activities (e.g. Quality improvement)	2
	Training	1
Patients	Making a difference	8
	Diversity	5
	Complexity	4
	Knowing patients well	4
	Time to help	3
Knowledge	Holistic approach	6
	Academic challenge	4
	Mental health overall importance	3
	Physical and mental health	2

### Prior experience

Psychiatrists listed both prior positive and negative views of psychiatry as factors affecting the decision to choose the specialty. Positive experiences of psychiatry as an undergraduate and a postgraduate also featured prominently:

‘I was not sure what kind of doctor I wanted to be when I was at medical school. But within a couple of weeks of starting my psychiatry placement, I knew psychiatry was for me.’

‘As a medical student undecided about my future career I met a lady, an older lady, with a terrible psychosis which had robbed her of her life and of her family. But I saw that with the help of a very compassionate psychiatrist and an amazing team, how she got her life back. And then I decided that was for me.’

Role models were also noted to be influential in the decision to choose psychiatry.

### Career factors

A number of psychiatrists spoke about having high job satisfaction, and having no regrets about their decision:

‘Psychiatry is a career which has enriched my life immeasurably’

‘I have not regretted a single moment in the last 20 years working as a psychiatrist.’

The variety of the job was noted as a positive factor:

‘As a psychiatrist I found that I can be a scholar, but also a communicator, a negotiator, an activist, an advocate, an educator. I often play the role of being a doctor, but sometimes I have to think like a psychologist, a sociologist, a social worker or even a detective.’

Having a role in teaching, and receiving good training were also mentioned as sub themes, as well as the ability to take part in additional activities (such as quality improvement).

‘I train trainees and I work with students and I do a lot of teaching, and that is an amazing part of my job.’

Working with a good team was also noted to be a positive reason to choose psychiatry.

### Patient factors

A number of participants spoke about working with patients, being interested by both the complexity and the diversity of people they see:

‘I get so much job satisfaction helping people to deal with complexities of their problem.’

‘I got to learn that it’s the patient and not the illness or the presenting problem that’s the most interesting part about this form of medicine.’

Having time to help and getting to know patients well also featured:

‘Yes we have time, but it takes time to get to know patients, it takes time to get to know them, hear their stories’

‘For those who like the idea of spending more time face to face with your patients, this is the specialty for you to consider.’

Feeling like you could make a difference was a significant sub theme:

‘I feel that the difference that one can make as a psychiatrist is astounding’

### Knowledge factors

The holistic approach taken in psychiatry was noted by participants:

‘Also the emphasis on the bio-psycho-social model, which seemed to make an awful lot of common sense. If we truly want our patients to get well and stay well then that really requires a holistic management plan.’

The academic challenge of psychiatry, and the relationship between physical and mental health also featured:

‘I understood a lot more about the complexity of the brain and how what bizarre problems could arise from difficulties with the brain and that convinced me, psychiatry was the thing for me.’

The overall importance and significance of mental health was mentioned as well:

‘There is no health without mental health’

## Discussion

The reasons why each doctor chooses to specialise in psychiatry will be personal to them, but there are common themes presented in the videos posted on Twitter as a part of #the60secondchallenge which are worthy of further consideration. These included an individual’s prior experience of psychiatry as well as positive factors of working in the specialty. There was engagement with these videos on Twitter, which may suggest it being a useful platform for involving a broad range of voices in the Choose Psychiatry campaign. However, the authors highlight this may not necessarily be the target audience for the campaign.

### Prior experience

Previous positive views, positive experience and exposure to positive role models in both the undergraduate and postgraduate years were identified as being favourable factors. Whereas negative views influenced by denigration in other specialties was identified as an initial deterrent, which was later overcome through exposure and positive experience. Positive views prior to the undergraduate years was not identified as having had an impact in these videos, although the positive views of sixth-formers regarding psychiatry tend not to be maintained throughout the undergraduate years in any case ( Maidment *et al.*, 2003; Choudry and Farooq, 2017). This highlights the time spent in undergraduate training as particularly influential ( Eagles *et al.*, 2007).

The imbalance and over-emphasis on acute hospital based medicine compared with psychiatry within the undergraduate years has already been identified as a disincentiveto choosing psychiatry ( Whitcomb and Cohen, 2004). This limits exposure to psychiatry as an early career influencer and exposes medical students early in their training to denigration of the specialty ( Creed and Goldberg, 1987; Howe and Ives, 2001; Littlewood, Ypinazar and Margolis, 2005; Curtis-Barton and Eagles, 2011; Ajaz *et al.*, 2016).  On the back foot, this necessitates that the short time spent within psychiatry needs to be positive within both clinical and taught settings ( McParland *et al.*, 2003; Choudry and Farooq, 2017). This offers further support for the recommendations to include enrichment activities, in addition to continuing the efforts of the ‘Anti-BASH’ campaign supported by the College ( Mukherjee, Maier and Wessely, 2013; Choudry and Farooq, 2017; Mortlock *et al.*, 2017; The Royal College of Psychiatrists, 2017a).

Positive experiences within the postgraduate years is also identified as a determinant, with the majority of doctors choosing to specialise in psychiatry after graduation ( Brown, Addie and Eagles, 2007; Dein, Livingston and Bench, 2007; Choudry and Farooq, 2017). This may be especially important in offering a remedy for negative views or career deterrents established earlier in training.  This supports the ongoing efforts targeting exposure and opportunities in psychiatry for foundation year doctors, and further highlights the importance of targeting psychiatry bashing by other specialties ( Kelley, Brown and Carney, 2013; Mukherjee, Maier and Wessely, 2013; Ajaz *et al.*, 2016; Boyle *et al.*, 2016; The Royal College of Psychiatrists, 2017c).

The well-known impact of positive role models on career specialty choice was also mentioned through these videos ( Goldacre *et al.*, 2005; Archdall, Atapattu and Anderson, 2013). Undergraduate teachersand postgraduate trainers may have a particular role to play here ( McParland *et al.*, 2003). However, it serves as a reminder for all psychiatrists to assume responsibility and encourages the involvement of everyone who wishes to promote psychiatry as a specialty choice to participate in opportunities for contact with undergraduate medical students and junior doctors.

### Career factors

Job satisfaction was one of the strongest subthemes to emerge from the videos with people discussing how they enjoyed the specialty or how the specialty had enriched their life in some way.  This may be particularly advantageous as a ‘selling point’ given its inference towards perceived favourable job conditions which have been identified as one of the top reasons for junior doctors choosing psychiatry ( Dein, Livingston and Bench, 2007; Curtis-Barton and Eagles, 2011; Denman, Oyebode and Greening, 2016). They are also one of the most important factors in a medical student’s choice of future career ( Dorsey, Jarjoura and Rutecki, 2003; Lefevre *et al.*, 2010).  The expectation of the working hours being more compatible with family life, alongside better working conditions and quality of life compared to other specialties has been particularly identified as favourable influencing factors ( Goldacre *et al.*, 2005; Dein, Livingston and Bench, 2007; Curtis-Barton and Eagles, 2011).

Although not an area explored by the twitter videos it may be important to note here that recently the influence of such ‘controllable lifestyle factors’ have become more influential compared to previous factors such as income and prestige. With controllable lifestyle defined by personal time free of practice requirements for leisure and family pursuits, and control of the total weekly hours spent on professional responsibilities ( Dorsey, Jarjoura and Rutecki, 2003; Lefevre *et al.*, 2010). However, as other specialties align with new working hour contracts and psychiatry is called to offer an expanding service this may not remain a feature specific to the specialty.  This change in motivating factors from traditional factors to specific lifestyle-based factors would be well remembered in future #ChoosePsychiatry campaigns to ensure targeting of the factors that we know motivate students the most.

Job variety and opportunities were also identified as positive career related experiences for choosing psychiatry, to include teaching and supplementary activities such as quality improvement projects.  Being the experience of those having chosen psychiatry may be indicative of its value here, but it may also be a positive sign of career opportunities having changed over the years in response to previously reported unfavourable factors.  Limited options have been reported in the past and identified as serving as a barrier to making psychiatry a career choice in the long-term, including poor job variety and research opportunities ( Barras and Harris, 2012; Archdall, Atapattu and Anderson, 2013).  Perhaps further supporting psychiatry’s apparent responsiveness to past reports, trainees have also previously reported poor supervision as a reason for not pursuing a choice in psychiatry, but this was identified as having been a positive experience through this study ( Choudry and Farooq, 2017).

The opportunity for the campaign to offer further mitigation of negatively held views regarding a career in psychiatry can be seen here. Through offering a platform for psychiatrists to voice their own experiences this enables a challenge to such potentially misconceived ideas about the specialty. The study’s authors consider this to be a particular encouraging feature of the specialty, that it appears able to respond to its previously identified barriers and to positively change experiences for its trainees, and is a worthy highlight of such campaigns.

### Patient related experiences

Some of the most important reasons identified by previous studies for choosing psychiatry is a doctor’s empathy for patients with mental illness, an interest in people and a sense of fulfillment in seeing people get better ( Dein, Livingston and Bench, 2007; Denman, Oyebode and Greening, 2016).  This is supported by the themes emerging from this study with themes about wanting to make a positive difference to the lives of patients with mental illness and a focus about the patient and having the opportunity to get to know them well being apparent.  Experiences with patients with mental illness were further identified as being particularly rewarding given the diversity and complexities of their presentations, and the specialty being identified as supportive here in enabling enough time to effectively help patients. However, this more personable interaction, despite being a positive by those already dedicated to the specialty, may be perceived as unfavourable by others to whom it may imply a lack of scientific basis and clinical approach ( Choudry and Farooq, 2017).  The limited psychiatric clinical exposure of medical students and foundation doctors also appears to offer a challenge here, leading to misconceptions that patients with mental illness are somewhat incurable and challenging as opposed to promoting the positive experiences reported by those having chosen psychiatry ( Dein, Livingston and Bench, 2007; Curtis-Barton and Eagles, 2011).

#### Knowledge

A recurring theme to emerge is the opportunity afforded by psychiatry to offer a wide-ranging and holistic treatment approach for a patient.  This is something that has been reported as having been particularly identified in the postgraduate years and is identified as one of the top shared factors for core trainees and consultants choosing psychiatry ( Dein, Livingston and Bench, 2007; Denman, Oyebode and Greening, 2016). This holistic approach has also been identified as beneficial for doctors regardless of career choice, with doctors appreciating that mental illness is encountered in all specialties and that psychiatric clinical experience offers the opportunity to gain transferable skills to enable management of the ‘whole’ patient ( Boyle *et al.*, 2016). This supports the premise that more doctors should have exposure to psychiatric placements regardless of an aim for recruitment, albeit that this may also favourably influence the number of trainees opting to choose psychiatry. Another example of this identified through the videos involves the description of psychiatry’s interface with physical health concerns, such as medically unexplained symptoms – this is an area previously cited as being limited and a barrier to trainees choosing psychiatry ( Barras and Harris, 2012; Archdall, Atapattu and Anderson, 2013).

Finding the specialty intellectually challenging and enjoyable to this end is a commonly identified feature, with a trainee’s self-perceived aptitude to manage this being a favourable factor influencing choice ( Denman, Oyebode and Greening, 2016; Choudry and Farooq, 2017).  However this appears to be more readily identified in retrospect by Consultants and may be less important in ‘selling’ the choice to a junior population where it seems it may not necessarily mitigate against more unfavourable factors. For example, psychiatry is already considered an interesting and intellectually stimulating topic but still not an attractive career choice ( Malhi *et al.*, 2003).

#### Limitations

This study considered the videos that were voluntarily put online by psychiatrists in response to a Twitter campaign inviting people to take part and as such a self-selection bias is possible.  It is likely that those participating in the campaign were interested in promoting psychiatry and were particularly keen to promote it in a positive fashion and to make their contribution socially desirable, which will have likely further biased its content in this regard.  The detail provided in the videos is also likely subject to recall biases given that the participants had already chosen psychiatry and were likely some years into their specialty choice.

The fact that no direct questioning was used to script the videos suggests that the videos are more likely to reveal the individual’s real and personal reasons for choosing psychiatry, although the campaign’s challenge to do this in sixty seconds probably reduced opportunity to express this fully.  The fact that this campaign was conducted on social media also restricts its invitation to participants and therefore offers a skewed and minority opinion, which may not be generalisable to the wider population of the specialty. This also raises the question of who the videos will be seen by, with a number of the online interactions with the videos coming from Twitter users who identify as already being psychiatrists or accounts associated with a company or group of people who the campaign was not intending to address.

Similarly, one of the difficulties with the data from these videos is that it is being offered by doctors who have already chosen psychiatry. In considering this, it may be that the identified themes do not add to the opportunity to improve recruitment but rather simply detail the thoughts of those people who were already pre-determined to become psychiatrists. However, the authors feel that exploring positive factors these doctors have identified can contribute to the recruitment of those who may not be future psychiatrists already.

## Conclusion

Time spent in both undergraduate and postgraduate training influence a doctor’s decision to choose psychiatry.  Clinical experiences appear particularly important in challengingthe misconceptions and career deterrents encountered by medical students and which can favourably influence a doctor to choose psychiatry despite this.  By ensuring the provision of postgraduate training posts, such as during the foundation years, there is an opportunity to offer trainees greater psychiatric clinical exposure and the opportunity to experience the positive aspects of the specialty and its patients’ recovery. Training posts also provide doctors who do not choose psychiatry an opportunity to develop transferable skills to support the quality of care they will deliver to their own patients, including their patients with mental illness.

Perceived job satisfaction, an opportunity to deliver holistic care and a sense of making a difference through working in psychiatry appear to have been particularly influential in making the decision to choose psychiatry for participants. Continued review of the changing desires of the medical workforce and the promotion of such possibilities from those whom choose psychiatry is required though, as reported barriers and aids appear to fluctuate.  It may be beneficial for the specialty to more directly emphasise its response so far to the barriers previously cited by trainees and to promote itself as a responsive and evolving career choice.

Regarding the #ChoosePsychiatry campaign itself, the content of these videos are supportive of its key messages and further unscripted engagement with psychiatrists at the periphery of the campaign may be supplementary to its aims.  However, further consideration of the social media platforms that the campaign’s target audience engage with would be beneficial, ensuring that it reaches those it intends to and encouraging psychiatrists who are keen to engage with such campaigns to target more relevant social media platforms, which may be outside of Twitter. The authors note that the Royal College of Psychiatrists has subsequently set up an Instagram account, one example of the broad range of platforms available.

## Take Home Messages

• Twitter has been used as a medium for peripheral engagement with the #ChoosePsychiatry campaign, through #the60secondchallenge videos.
• Four main themes were identified in the reasons for choosing psychiatry: prior experience of psychiatry, career factors, patient factors and knowledge factors.
• Undergraduate and postgraduate experiences, job satisfaction, an opportunity to deliver holistic care and a sense of making a difference were influential factors in choosing psychiatry.
• Findings here support efforts to improve the undergraduate experience and to increase foundation posts, as well as maintaining some of the current key messages of the campaign.
• Further consideration of the most appropriate social media platform to ensure engagement with the target audience of campaigns like this may be helpful.

## Notes On Contributors

Dr Jonathan Cunliffe is a junior doctor currently working in psychiatry at Sheffield Health and Social Care NHS Foundation Trust, with an honorary role at The Medical School, The University of Sheffield. ORCID: https://orcid.org/0000-0002-4696-6578

Dr William Melton is a junior doctor working in South Yorkshire, with an interest in Medical Education.

Dr Helen Hargreaves is a psychiatry registrar with an interest in Medical Education, working at Northumberland, Tyne and Wear NHS Foundation Trust.

Dr Reem Abed is a consultant psychiatrist at Sheffield Health and Social Care NHS Foundation Trust.

## Declarations

HH and RA uploaded videos to Twitter as part of #the60secondchallenge. They were not directly involved in the analysis of their own videos.
