# Supplementary material for: #ChoosePsychiatry #the60secondchallenge – a thematic analysis of videos published on Twitter about reasons for choosing psychiatry
Source: MedEdPublish (2016). 2018 Nov 16;7:261. [Version 1] doi: 10.15694/mep.2018.0000261.1 (PMC10711973; doi:10.15694/mep.2018.0000261.1)
Supplement: Peer review history

# Reviewer report: Reviewer response for version 1

**DOI:** 10.21956/mep.19717.r29516

**Published:** 2018-12-01

**Authors:** Johnny Lyon Maris

**Affiliations:** 1 Southampton GP Education Unit

This review has been migrated. The reviewer awarded 3 stars out of 5 Thank you for uploading this mainly descriptive piece that is well referenced and written. It adds to the literature around social media campaigns and highlights specialities that need promotion. A link to the videos would have enhanced it and is the beauty of publication onto the web. Is Twitter the main platform used by undergraduates and postgraduate trainees? If it is not then the power of the medium will be reduced.

Reviewer Expertise:

NA

No decision status is available

# Reviewer report: Reviewer response for version 1

**DOI:** 10.21956/mep.19717.r29515

**Published:** 2018-11-26

**Authors:** Mark Hanson

**Affiliations:** 1 Hospital for Sick Children

This review has been migrated. The reviewer awarded 3 stars out of 5 This paper speaks to the innovative potential that social media may represent within recruitment and selection to postgraduate psychiatric training. In this study paper, a thematic analysis is undertaken of twitter videos of psychiatrists describing their motivation for a psychiatric a career. These videos were part of a recruitment strategy to increase the numbers of psychiatric trainees and ultimately psychiatrists. The analysis of the psychiatry videos outlining motivations and factors associated with the selection of a career in psychiatry reinforce the literature in this area. The particularly interesting and innovative aspect of the study is its identification of social media as means to target prospective psychiatry trainees (medical students and Foundation doctors). The author’s report that there was a subsequent increase in recruitment into psychiatry but can only speculate upon the impact this social media approach may have actually had upon it. Future selection research should evaluate the link between use of social media, recruitment and selection into Psychiatry and other specialties.

Reviewer Expertise:

NA

No decision status is available

# Reviewer report: Reviewer response for version 1

**DOI:** 10.21956/mep.19717.r29512

**Published:** 2018-11-25

**Authors:** John Cookson

**Affiliations:** 1 University of Worcester

This review has been migrated. The reviewer awarded 4 stars out of 5 I agree with the other comments; as so often a positive experience as an undergraduate is a key determinant. It would be interesting to know what makes for a postivie experience but I would anticipate that the focus on the community rather than the hospital would be important. Perhaps we now need a survey of student attitudes

Reviewer Expertise:

NA

No decision status is available

# Reviewer report: Reviewer response for version 1

**DOI:** 10.21956/mep.19717.r29513

**Published:** 2018-11-17

**Authors:** Ken Masters

**Affiliations:** 1 Sultan Qaboos University

This review has been migrated. The reviewer awarded 3 stars out of 5 A well thought out and written paper. The background is clearly stated, the data are displayed, and the discussion grounds the results very nicely in the literature. The limitations are objectively noted - and may even have been a little over-stated, as the idea was to discuss the reasons given, not necessarily the validity of the reasons. If there are problems with the videos (e.g. bias), then that may be a problem with the data, but not with the research. My only complaint is that the authors should have identified (and linked to) the actual videos, in much the same way that one identifies individual papers in a literature review. One of the problems with so much research is that readers cannot get access to the raw data. This is usually because of confidentiality issues. In this case, however, the videos are publicly available, so links to the raw data could have been provided (otherwise readers have to manually perform the search). In addition, if the authors had done this, it may have given them an opportunity for some more detailed analysis of the results, much in the way that a systematic literature review is performed.I would strongly recommend that the authors provide a revised version of their paper in which they:• Identify and link to the videos• Have a second table in which they provide some commentary on each of the videos.

Reviewer Expertise:

NA

No decision status is available

# Reviewer report: Reviewer response for version 1

**DOI:** 10.21956/mep.19717.r29514

**Published:** 2018-11-17

**Authors:** Balakrishnan Nair

**Affiliations:** 1 Centre for Medical Professional Development and University of Newcastle

This review has been migrated. The reviewer awarded 4 stars out of 5 This is an interesting study using innovative methods to recruit the new generation to specialty training. Medical Educators need to reflect on what the new generation of students and doctors need for their learning and training and come up with innovative ways to do this . This is the only way to attract them to specialties where they are needed for the benefit of the population health needs . . The millennials are more likely to engage through social media and this study is a good example of using this methodology This study highlights the value of junior doctors coming up with new ways of doing research ; they know what their peers want and need.The conclusions are well known to medical educationalists. Positive experience and good role models have positive influence on career choice. Once you become a specialist , one gets more job satisfaction from work – life balance , connecting with patients with a meaningful long term partnership with them . If you can communicate this with the younger generation, they are more likely to be attracted your specialty

Reviewer Expertise:

NA

No decision status is available
